# Comparative analysis of dental pulp stem cells and stem cells from human exfoliated teeth in terms of growth kinetics, immunophenotype, self-renewal and multi lineage differentiation potential for future perspective of calcified tissue regeneration

**DOI:** 10.12669/pjms.38.5.5187

**Published:** 2022

**Authors:** Shagufta Naz, Farhan Raza Khan, Irfan Khan, Raheela Rahmat Zohra, Asmat Salim, Nuruddin Mohammed, Tashfeen Ahmad

**Affiliations:** 1 Ms. Shagufta Naz, M.Sc. Department of Biotechnology, University of Karachi, Pakistan. Department of Surgery, Aga Khan University, Karachi, Pakistan; 2 Dr. Farhan Raza Khan, FCPS. Department of Surgery, Aga Khan University, Karachi, Pakistan; 3 Dr. Irfan Khan, Ph.D, Dr. Panjwani Center for Molecular Medicine and Drug Research, International Center for Chemical and Biological Sciences, University of Karachi, Pakistan; 4 Dr. Raheela Rahmat Zohra, Ph.D. Department of Biotechnology, University of Karachi, Pakistan; 5 Dr. Asmat Salim, Ph.D, Dr. Panjwani Center for Molecular Medicine and Drug Research, International Center for Chemical and Biological Sciences, University of Karachi, Pakistan; 6 Dr. Nuruddin Mohammed, PhD, FMFM Department of Obstetrics and Gynecology, Aga Khan University, Karachi, Pakistan; 7 Dr. Tashfeen Ahmad, FCPS, Ph.D. Departments of Surgery and Biological & Biomedical Sciences, Aga Khan University, Karachi, Pakistan

**Keywords:** DPSC, SHED, Colony forming units, Population doubling time, Tri-lineage differentiation

## Abstract

**Background and Objectives:**

Owing to high proliferation rate, multipotency and self-renewal capability, dental pulp stem cells (DPSC) and stem cells from human exfoliated teeth (SHED) have become stem cell source of choice for cell based regenerative therapies. We aimed to compare DPSC and SHED as stem cell sources with a future use in regeneration of calcified tissue.

**Methods:**

Explant derived human DPSC (n=9) and SHED (n=1) were cryopreserved, thawed and expanded for analysis of population doubling time, colony forming unit assay and efficiency. A growth curve was plotted to determine population doubling time, while colony forming numbers and efficiency was determined at plating cell densities of 5.6, 11.1 and 22.2 / cm2. The isolated cells were characterized for the presence of stem cell markers by immunophenotyping and immunofluorescence staining, and tri-lineage differentiation. Statistical analysis was performed by Pearson correlation, Exponential regression and two way Anova with Tukey test at p<0.05.

**Results:**

DPSC and SHED exhibited spindle shaped fibroblast like morphology. SHED was found superior than DPSC in terms of proliferation and colony forming efficiency. Immunophenotypes showed that DPSC contain 62.6±26.3 %, 90.9±14.8% and 19.8±0.1%, while SHED contain 90.5%, 97.7% and 0.1% positive cells for CD90, CD73 and CD105. DPSC were strongly positive for vimentin, CD29, CD73, while reactivity was moderate to weak against CD44 and CD90. SHED expressed vimentin, CD29, CD105, CD90 and CD44. Both were negative for CD45. Upon induction, both cell types differentiated into bone, fat and cartilage like cells.

**Conclusion:**

Cultured DPSC and SHED were proliferative and exhibited self-renewal property. Both DPSC and SHED expressed stem cell markers and were able to differentiate into bone, fat and cartilage like cells. Thus, these could be a suitable stem cell sources for cell based regenerative therapies.

## INTRODUCTION

Loss of bone and dental tissues due to disease, trauma or surgery have become a global concern due to its adverse effect on individual’s wellbeing and economic burden on affected individuals.^1^ At present, the gold standard therapy for bone defects is autologous bone transplant. However, due to donor site morbidity and difficulty in harvesting graft from donor site, use of autologous bone is restricted.^2^ Regarding tooth tissue, dental implants, prostheses^3^ and periodontal treatments^4^ are available options for dental problems. Such therapies are only meant to stop disease progression and improve clinical condition; they mostly fail to regenerate lost tissue. Regenerative medicine is still in its infancy to promise any predictable solution for regenerating lost tissues.^1^

Owing to their capability for tissue regeneration, dental mesenchymal stem cells (MSC) have gained a lot of attention. Identified nearly two decades ago, dental pulp stem cells (DPSC) and stem cells from human exfoliated teeth (SHED) have proven to be promising stem cell choices for cell based regenerative therapies. Though isolated from dental pulp, these are not restricted to regenerating tissues of dental origin such as pulp-dentin complex^5^ and regeneration of alveolar bone to repair periodontal defect.^6^ Aptitude to cross lineage boundaries is already established thus proving their utility in regenerating tissue types other than dental tissue.

Having enormous regenerative potential, both DPSC and SHED have already made their ways to clinical translation. Autologus DPSC were used to successfully treat irreversible pulpitis demonstrating no toxic effect with positive electric pulp and similar magnetic resonance imaging (MRI) intensity to normal pulp indicating complete regeneration of dental pulp.^5^ In a randomized clinical trial, autologous SHED were demonstrated to regenerate dental pulp with sensory nerves and blood vessels in traumatic teeth of children.^7^

The present study is intended to compare two cryopreserved dental stem cell types DPSC and SHED from dental pulps of permanent and primary teeth respectively. The proliferation of both stem cell types was determined using population doubling time. Colony forming units at low cell density showed self-renewal of both DPSC and SHED. The expression of stem cell markers were assessed by immunophenotyping and immunofluorescence staining. Further, induction of DPSC and SHED showed that these can differentiate into bones, cartilage and fat like cells. We found that both DPSC and SHED could be an autologous cell sources for regenerating calcified tissue.

## METHODS

### Collection and Processing of Human Tooth Samples:

Human permanent teeth from healthy adults (32±11 years) and naturally exfoliated primary teeth (12±0 years) were collected from individuals / subject / patients at dental clinics at Aga Khan University Hospital, Karachi, during February 2016 to June, 2017, under approved guidelines set by institutional Ethics Review Committee (ERC), Ref. Number 4-1997-BBS-ERC-12. This study was performed according to Helsinki declaration. Written consents (or assents) were obtained from participants who donated their teeth already due for extraction. Teeth samples were processed within 24 hours of extraction.

### Culture of Dental MSCs:

Explant culture of human dental pulp derived MSC was performed according to our previous published protocol.^8^ Briefly, pulps were extirpated, treated with antibiotic antimycotic solution (penicillin 100U/mL, streptomycin 100μg/mL, amphotericin B 0.25μg/mL), rinsed in 1X phosphate buffered saline (PBS) (Sigma-Aldrich, Inc, USA) and minced into approximately 1–2 mm^3^ pieces using surgical blade. Minced fragments were cultured in Dulbecco’s modified essential medium F12 (DMEM-F12) (Sigma-Aldrich, Inc, USA) supplemented with 20% fetal bovine serum (FBS), penicillin 100U/mL, streptomycin 100μg/mL, amphotericin B 0.25μg/mL, 1mM sodium pyruvate and 2mM L-glutamine (Sigma-Aldrich, Inc, USA). DPSC and SHED were cryopreserved (90% FBS and 10% dimethyl sulfoxide (DMSO) (Sigma-Aldrich, Inc., USA) in liquid nitrogen after establishing primary culture and were subsequently thawed and expanded for later use.

### Population Doubling Time (PDT):

To determine growth rate, DPSC and SHED were seeded into 24-well culture plates (Thermo fisher Scientific, US) at cell densities of 1x10^4^ and 3×10^4^ cells/per well. Cell number was assessed every day for 12 days (2 replicates for each time point). Cells were stained by trypan blue (Sigma-Aldrich, Inc, USA). Unstained cells were counted using hemocytometer (Marienfeld, Germany) under light microscope (IX70, Olympus). PDT was calculated from log phase of growth curve. An exponential trend line was used to calculate growth rate. R-squared value was also obtained. Using an exponential regression equation curve of best fit was obtained along with equation amount. PDT was calculated using formula: PDT = ln2 / k where, k = growth rate (constant) and ln2 = 0.693.

### Colony Forming Unit Assay (CFU):

DPSC and SHED were seeded at cell densities ranging from 5.6, 11.1 and 22.2 cells / cm^2^ in 35 mm plate (Corning, US) (duplicate). After seeding, plates were incubated at 37°C with 5% CO_2_. At day 14, cells were fixed with 4% paraformaldehyde (Merck, US) for 15 minutes and stained with 3% crystal violet (Merck, US) for one hour at room temperature. Stained colonies were counted microscopically (IX70, Olympus) and macroscopically. For each sample, colonies containing more than 50 cells were included. Colony forming unit efficiency (%) was calculated using formula: mean number of colonies / total number of cells seeded x 100. At mean cell density of 41.67cells/cm^2^ colonies were found overlapped thus no counting could be performed.

### Immunofluorescence Staining:

DPSC and SHED were fixed with 4% paraformaldehyde (Merck, US) for 10 minutes, permeabilized for 10 minutes with 0.5% Triton X-100 (Sigma-Aldrich, Inc, USA) and blocked in 2% bovine serum albumin (BSA) (Sigma-Aldrich, Inc, USA) for 1 hour at room temperature. Cells were subjected to mouse anti-human primary antibodies CD29 (Chemicon International, USA) CD44, CD73, CD105, CD45 (BD Pharmingen, USA), vimentin (Sigma-Aldrich, Inc, USA), CD90 (EMD Millipore Corp, USA) using manufacturer recommended dilutions at 4ºC overnight followed by incubation with Alexa Fluor 488 or 546 conjugated goat anti-mouse secondary antibody (Invitrogen, US) at 1:200 dilutions at 37°C for one hour. Nuclei were counterstained with DAPI (MP-Biomedical, Inc, USA) at 1 μg/mL in 1X PBS for 10 minutes at room temperature. Images were captured using a Ti2 inverted microscope (NIKON, Japan) using acquisition software NIS-Element AR.

### Immunophenotyping:

Flow cytometry was used to assess cell surface marker profile. Briefly, DPSC and SHED were grown to 70% confluency, detached, washed with 1X PBS and centrifuged to pellet down and blocked with 1% BSA (Sigma). Staining was performed as per manufacturer’s (human MSC immunophenotyping kit, 130-095-198 Miltenyi) instructions. Briefly, 5.0 x 10^5^ cells were stained with a cocktail of antibodies; Fluorescein isothiocyanate (FITC) conjugated CD90, Allophycocyanin (APC) conjugated CD73, Phycoerythrin (PE) conjugated CD105, Peridinin Chlorophyll Protein Complex (PerCP) conjugated; CD34, CD45, CD14 and CD20. Cells were analyzed on a Fluorescence activated cell sorter (FACS) Canto II (Becton Dickinson, US) using Diva Software (Beckton Dickinson). Cells were analyzed as 10,000 events for each sample.

### Tri-Lineage Differentiation:

DPSC and SHED were chemically induced to differentiate along chondrogenic, osteogenic, and adipogenic lineages by culturing monolayers of DPSC and SHED in specific differentiation medium for three weeks. Chondrogenic differentiation was induced using chondrogenic induction medium containing 20 ng Transforming growth factor beta-1 (TGFβ1) (Merck, Millipore), 10 ng insulin, 100 nM dexamethasone (Sigma-Aldrich, Inc., USA) and 100 μM ascorbic acid (Dae-Jung Chem and Metal Co, Korea). Cells were stained with 1% toluidine blue to detect extracellular matrix produced by chondrogenic derivatives. Osteogenic differentiation was induced by using osteogenic medium containing 0.1 μM dexamethasone (Serva, GmbH), 10 μM β-glycerophosphate (Sigma Chemical Corp, USA) and 50 μM ascorbate phosphate (Dae-Jung Chem and Metal Co, Korea). Cells were stained with Alizarin Red S stain (Sigma-Aldrich, Inc., USA) to visualize calcium deposition. Adipogenic differentiation was induced using adipogenic medium containing 0.5 μM isobutyl-methylxanthine (Sigma-Aldrich, Inc, USA), 1 μM dexamethasone (Serva, GmbH), 10 μM insulin (Sigma-Aldrich, Inc., USA), and 200 μM indomethacin (MP Biomedical). Cells were stained with Oil Red O (Sigma-Aldrich, Inc., USA) to determine presence of lipid droplets. Cells grown in regular media served as negative controls.

### Statistical Analysis:

Data were represented as mean and standard error of mean (SEM). Growth curve data was analyzed using Pearson correlation and Exponential regression. Log phase of growth curve was plotted, and compared with regression line. It showed positive relationship between days and number of cells. Two way Anova analysis with with Tukey test was used to analyze colony forming unit assay at p < 0.05.

## RESULTS

### Culture of Dental Pulp Stem Cells:

**Fig.1:**
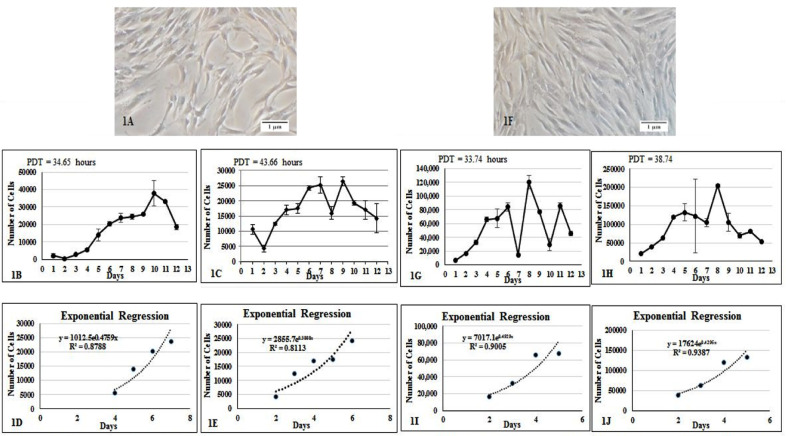
Primary culture established from permanent and primary teeth. (1A) DPSC obtained from maxillary third molar and (1F) SHED from primary teeth. Both cell types exhibit characteristic spindle shaped fibroblast like morphology. Proliferation of DPSC. Growth curve of DPSC at (1B) 10,000 and (1C) 30,000 density. Graph showing estimation exponential regression curve for DPSC at (1D) 10,000 and (1E) 30,000 cell density. Proliferation of SHED at (1G) 10,000 and (1H) 30,000 cell density. Graph showing estimation of exponential regression curves for SHED at (1I) 10,000 and (1J) 30,000 cell density. The readings appeared above showed log phase of growth curve. The X axis shows time in days, and Y axis represents the number of cells. Error bars represent SEM.

Obtained DPSC and SHED were adherent, exhibit fibroblast-like morphology suggestive of mesenchymal origin. There were no morphological differences between the cells obtained from deciduous teeth and impacted third molars (Fig.1A & 1F).

### Growth Curve and Population Doubling Time (PDT):

A short term *in vitro* culture was set up to assess growth / population kinetics of DPSC and SHED. A growth curve was plotted to assess cell proliferation of DPSC and SHED at two different cell densities i.e. 10,000 and 30,000 cells / well. Growth curve seemed to suggest that SHED have higher proliferation rate than DPSC. At 10,000 cell density, DPSC were found to grow exponentially at a growth rate of 0.4759 with an estimated PDT of 34.94 hours (Fig.1B & 1D), while at 30,000 cell density, the growth rate was 0.3808 with population doubling time of 43.66 hours (Fig.1C & 1E). At 10,000 cell density, SHED grew at a rate 0.4929 with an estimated PDT of 33.74 (Fig. 1G & 1I), while at 30,000 cell density the growth rate was found to be 0.4295 with an estimated PDT of 38.74 hours (Fig.1H & 1J).

### Colony Forming Efficiency (CFU):

**Fig.2:**
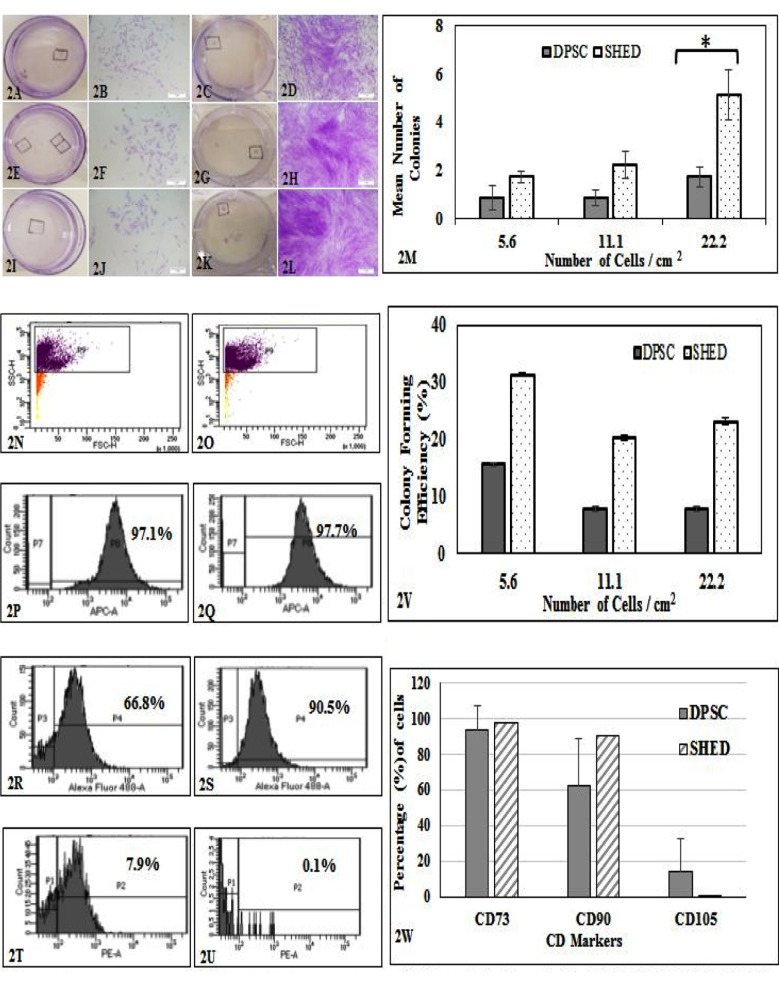
Colony forming Assay for DPSC and SHED. All 35mm plate showed colonies stained with crystal violet. (2A & 2C), (2E % 2G) & (2I & 2K) represent 5.6, 11.1, 22.2 cells / cm2 respectively. Representative colonies were marked in all plates and were imaged. Micrographs; (2B & 2D), (2F & 2H) and (2J & 2L) represent 5.6, 11.1 and 22.2 cells / cm2 respectively. Scale bar = 5μm. Graph (2M) compares number of colonies: ± showed significance at p<0.05, (2V) colony forming efficiency (%) between DPSC and SHED at plating cell densities of 5.6, 11.1 and 22.2 cells /cm2. Error bars represent SEM. Representative immunophenotypes of DPSC (left panel) & SHED (right panel). Histograms show presence of classical stem cell markers; (2N & 2O) show gated cells (horizontal box), (2P & 2Q) represent APC-CD73, (2R & 2S) represent FITC-CD90 and (2T & 2U) represent PE-CD105. (2W) Comparing percentage expression of classical stem cell markers..

Self-renewal was assessed by CFU assay. Colonies were observed in both cell types at all different plating densities used. Colony forming units were found to be comparatively higher in SHED (Figs. 2A, 2B, 2E, 2F, 2I & 2J) than DPSC (Figs. 2C, 2D, 2G, 2H, 2K & 2L). Mean number of colonies produced by DPSC was 0.88, 0.88, 1.75 and SHED was 1.75, 2.25, 5.13 over plating densities of 5.6, 11.1 and 22.2 cells per cm2 respectively (Fig. 2M). Colony forming efficiency (%) of SHED was found to be higher than DPSC (Fig. 2V). However, the difference was found to be statistically significant at 22.2 cells per cm2 (Fig.2M).

### Immunophenotyping:

DPSC and SHED were found to contain heterogeneous populations of stem cells expressing classical stem cell markers. Presence of markers were determined by flow cytometry and measured as percentages from gated population (Figs. 2N & 2O). DPSC contain 90.9±14.8%, 62.6±26.3% and 19.8±0.1% cells expressing CD73, CD90 and CD105 respectively (Figs.2P, 2R & 2T). SHED express 97.7% CD73, 90.5% CD90, and 0.1% CD105 cells (Fig. 2Q, 2S & 2U). We could not perform any descriptive statistics for SHED as there was only a single sample of primary teeth (Fig.2W).

### Immunofluorescence (IF) staining of classical MSC markers:

**Fig.3:**
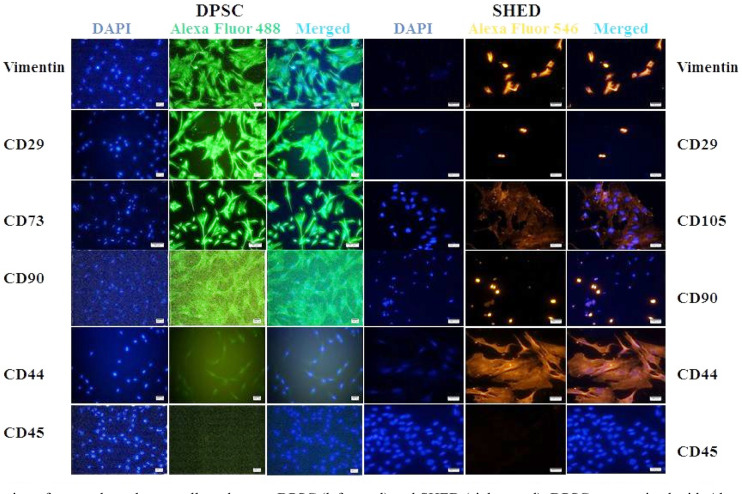
Expression of mesenchymal stem cell markers on DPSC (left panel) and SHED (right panel). DPSC stained with Alexa Fluor 488 (green) show the expression of vimentin, CD29, CD73, CD90, and CD44 in DPSC. SHED were stained with Alexa Fluor 546 (orange) show the expression of vimentin, CD29, CD105, CD90, and CD44. CD45 was used as negative marker. Micrographs were captured with Ti2 inverted microscope and analyzed using NIS element software. Scale bar= 100 μm and 50 μm for CD73 images only.

DPSC and SHED were further confirmed for their stem cell populations. DPSC (Fig.3 left panel) were strongly positive for vimentin, CD29, CD73, while moderate to weak reactivity was observed for CD44 and CD90. On the other hand, SHED (Fig.3 right panel) expressed vimentin, CD29, CD105, CD90 and CD44. Both DPSC and SHED were negative for CD45.

### Tri-Lineage Differentiation:

**Fig.4:**
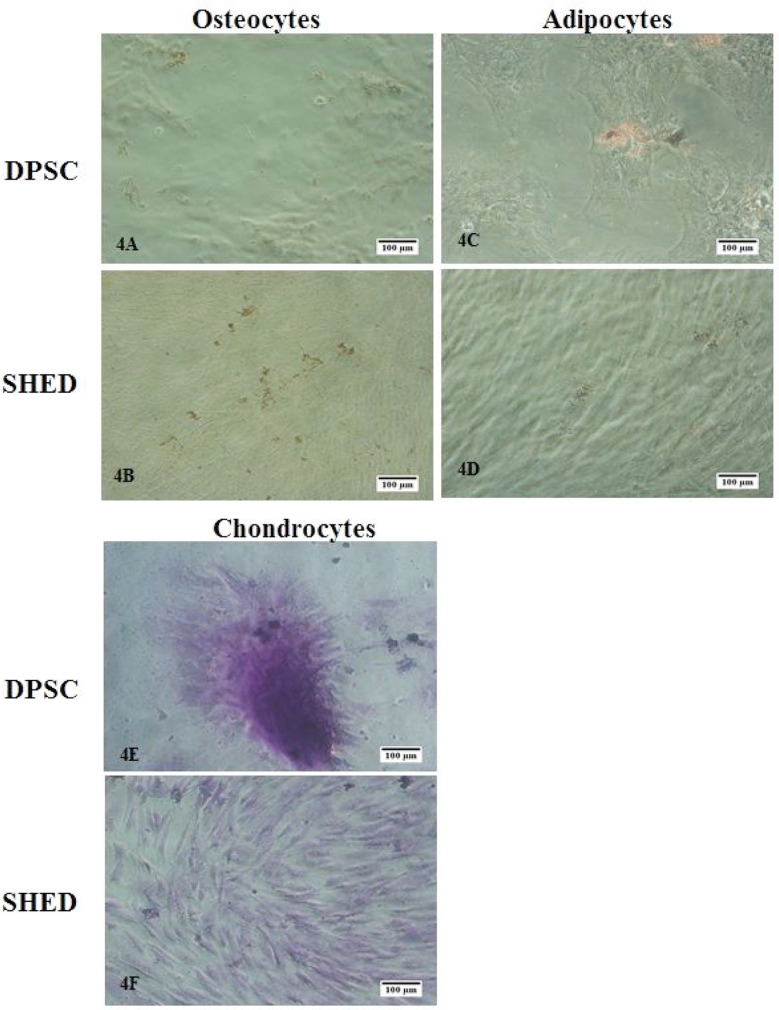
*In-vitro* tri-lineage differentiation assay. DPSC and SHED were induced to differentiate into; (4A & 4D) osteocytes like cells. Calcium deposits were detected by Alizarin Red S staining. (4B & 4E) Adipocytes like cells. Oil droplets produced by these cells were detected by Oil Red O stain. (4C & 4F) Chondrocytes like cells. Sulphated glycosaminoglycans (GAGs) produced by these cells were detected by staining with toluidine blue. Scale bar = 100μm.

After induction, DPSC and SHED were stained to detect presence of osteoblasts, adipocytes and chondrocytes like cells. Upon staining with Alizarin Red S, reddish brown colored calcium phosphate deposits were observed (Fig.4A & 4B). SHED were weakly positive for Oil Red O stain indicating low differentiation potential for adipocytes than DPSC (Fig.4C & 4D). Staining with toluidine blue demonstrated sulphated glycosaminoglycans (GAGs) produced by chondrocytes (Fig.4E & 4F).

## DISCUSSION

In this study, we aimed to compare two well-known stem cell types from dental origin. In culture, both DPSC and SHED exhibited similar morphology; fibroblast-like shape. We did observe epithelial like-cells in primary culture derived from supernumerary tooth from mixed dentition. However, these cells washed off during subsequent passages. As reported previously, DPSC and SHED are two distinct populations reflecting different developmental processes, tissue structure and function of permanent and primary teeth. SHED represents more immature population than DPSC, demonstrating higher proliferation rate and increased cell-population doublings.^9^ Our results were consistent to previous studies reporting a shorter PDT for SHED than DPSC^10^,^11^ confirming more mature phenotype of DPSC.

DPSC manifest higher proliferation rate than bone marrow MSC (BMMSC), a long considered source of MSC.^9^ Differently, SHED has advantage over DPSC, showing higher proliferation rate than DPSC^10^ and BMMSC.^12^ Self-renewal, a striking feature of stem cells, is assessed by colony forming unit assay. DPSC and SHED demonstrated good efficiency for colony formation. Nevertheless, SHED was found to be superior to DPSC. Also, SHED (79.5 ± 3.98%) produced significantly higher colony forming units than DPSC (47.6 ± 1.47%).^10^ Interestingly enough, significant variability in CFU efficiencies, growth curve and immunophenotyping profile were reported in stem cells from apical papilla from healthy donors of similar age and stage of tooth root development.^13^

To date there is no any single marker that can solely identify MSC. We employed other markers along with markers defined by Mesenchymal and Tissue Stem Cell Committee of International Society for Cellular Therapy (ISCT)^14^ to identify DPSC and SHED. As reported earlier in original publications, DPSC and SHED are comprised of heterogeneous populations and contain sub-populations of stem cells characterized by differential expression of cell surface markers. Our isolated SHED and DPSC were found to be positive for vimentin, CD29, CD90, CD73 and CD105, CD44, CD117. However, expression of these markers was variable among two cell types. These cells revealed negative staining for CD45 suggestive of absence from hematopoietic origin.

Remarkably, we observed two significant sub-populations CD105 ^low/ -^ and CD105 ^high/ +^ as previously reported in DPSC^15^ and SHED.^16^ A low or no expression of endoglin (CD105) was also observed in adipose tissue-derived MSC. These CD105^+^ MSC derived from adipose tissue (AT-MSC) were demonstrated to be favorably differentiated into chondrocytes^17^ whereas CD105^-^AT-MSC were more prone to osteogenic lineage differentiation.^18^ CD105^+^MSC derived from cord blood was demonstrated to have favorable pattern in infarcted heart.^19^ Careful evaluation of endoglin associated pathway suggested that improved osteogenesis among CD105-AT- was observed due to lower TGF-β1/Smad2 signaling in subpopulation. Thus, highlighting an important target area to enhance osteogenesis in adipose derived tissue for skeletal regeneration.^18^ Conversely, some studies report opposing outcomes and concluded that chondrogenic ability between CD105 populations from bone marrow MSC (BMSC) was not found significant. Expression of CD105 on expanded culture of BMSC does not correlate with chondroprogenitor phenotype suggestive of not choosing CD105 to isolate chondroprogenitor from BMSC population.^20^

Being multipotent, DPSC and SHED can reportedly be differentiated into three different lineages; osteoblasts, adipocytes and chondrocytes. This not only is considered as a routine test but also a salient feature of MSC. Upon induction, DPSC and SHED were found to be weakly stained with Alizarin Red S and Oil Red O thus requires further confirmation of differentiation at gene expression level. Toluidine blue did stain cells moderately. In a study, DPSC co-differentiated into osteocytes and endotheliocytes *in vitro* and *in vivo*.^21^ Regarding this, others also report proof-of-principle work by regenerating sub-cutaneous bone in rats using human adult DPSCs along with hydrogel scaffold.^22^ Human SHED demonstrated repairing maxillary alveolar defects in rats giving hope to repair human cleft lip and palate.^23^ We used monolayer cell culture for induction of chondrocytes. DPSC and SHED were stained with toluidine blue indicating production of sulphated glycosaminoglycans (GAGs). Implanted in rabbit knee, hDPSC subpopulation CD29+ CD44+ and CD105+ found to repair articular cartilage.^24^ DPSC in our lab, being positive for above mentioned markers may have future implication in cartilage regeneration. Comparing efficiency of DPSC, SHED and BMSC for regenerating 4mm calvaria defect in immunodeficient mice. Nakajima *et al*. found SHED to produce widely distributed collagen fibers and largest osteoid than DPSC and BMSC upon histological examination.^25^ Several reports confirm conservation of stemness in cryopreserved SHED^16^ and DPSC.^26^ We also used cryopreserved DPSC and SHED. However, we did not perform any comparative studies to investigate time period that these cells could retain cell surface markers, colony forming efficiency and multi-lineage differentiation.

### Limitations:

Although cultured DPSC and SHED fulfilled minimal recommended criteria required, a larger sample size would have permitted stronger statistical correlations. Molecular studies to detect expression of lineage specific genes for bone, fat and cartilage like tissue were not done due to budgetary limitations. Low proliferation and CFU of DPSC could be due to cellular replicative senescence, but confirming this requires further studies. More extensive *in vitro* studies will be required to further explore the usefulness and understanding of these stem cell types.

## CONCLUSIONS

 Results of this study show that DPSC and SHED exhibit proliferation, self-renewal and differentiation attributes required for regeneration of calcified tissue. SHED appear to exhibit better proliferation and self-renewal capability than DPSC. Presence of DPSC and SHED subpopulations may have future potential applications in regeneration of tooth, bone and cartilage. Transcriptional and translational profiles of these cell types may provide an in-depth insight for futuristic regenerative approaches.
